# The FAST technique: a simplified *Agrobacterium*-based transformation method for transient gene expression analysis in seedlings of Arabidopsis and other plant species

**DOI:** 10.1186/1746-4811-5-6

**Published:** 2009-05-20

**Authors:** Jian-Feng Li, Eunsook Park, Albrecht G von Arnim, Andreas Nebenführ

**Affiliations:** 1 Department of Biochemistry, Cellular and Molecular Biology, University of Tennessee, Knoxville, Tennessee 37996-0840, USA; 2 Current address: Department of Genetics, Harvard Medical School, and Department of Molecular Biology, Massachusetts General Hospital, Boston, Massachusetts 02114-2790, USA

## Abstract

**Background:**

Plant genome sequencing has resulted in the identification of a large number of uncharacterized genes. To investigate these unknown gene functions, several transient transformation systems have been developed as quick and convenient alternatives to the lengthy transgenic assay. These transient assays include biolistic bombardment, protoplast transfection and *Agrobacterium*-mediated transient transformation, each having advantages and disadvantages depending on the research purposes.

**Results:**

We present a novel transient assay based on cocultivation of young Arabidopsis (*Arabidopsis thaliana*) seedlings with *Agrobacterium tumefaciens *in the presence of a surfactant which does not require any dedicated equipment and can be carried out within one week from sowing seeds to protein analysis. This Fast Agro-mediated Seedling Transformation (FAST) was used successfully to express a wide variety of constructs driven by different promoters in Arabidopsis seedling cotyledons (but not roots) in diverse genetic backgrounds. Localizations of three previously uncharacterized proteins were identified by cotransformation with fluorescent organelle markers. The FAST procedure requires minimal handling of seedlings and was also adaptable for use in 96-well plates. The high transformation efficiency of the FAST procedure enabled protein detection from eight transformed seedlings by immunoblotting. Protein-protein interaction, in this case HY5 homodimerization, was readily detected in FAST-treated seedlings with Förster resonance energy transfer and bimolecular fluorescence complementation techniques. Initial tests demonstrated that the FAST procedure can also be applied to other dicot and monocot species, including tobacco, tomato, rice and switchgrass.

**Conclusion:**

The FAST system provides a rapid, efficient and economical assay of gene function in intact plants with minimal manual handling and without dedicated device. This method is potentially ideal for future automated high-throughput analysis.

## Background

Sequencing of the complete genomes of the model plant *Arabidopsis thaliana *and several other plant species has stressed the need to understand the functions of large numbers of unknown genes encoded within these genomes. Assuming that a gene function is generally attributable to biochemical activity, subcellular localization and interacting partners of its protein product, novel bioinformatic approaches have been able to annotate a large fraction of these uncharacterized genes [1]. However, experimental assays are required to confirm every *in silico *prediction or to resolve ambiguous or uncertain predictions. This means that a myriad of genes ultimately have to be expressed and then analyzed *in planta*. To fulfill this demanding task and gain reliable data, one has to count on a methodology which can express proteins in a simple and efficient manner in plants, particularly in Arabidopsis, under as close to normal physiological conditions as possible.

Stable Arabidopsis transgenic lines expressing epitope-tagged or otherwise modified genes offer advantages in terms of a sustainable supply of plant material with homologous protein expression, the potential of mutant complementation, as well as a global examination option throughout all tissues and cell types. Although the often used floral dip procedure [2] generates transgenic Arabidopsis plants with minimal labor, plants must still be grown to maturity over several weeks. The need to harvest seed and perform selection also makes it impractical to test large numbers of different transgene constructs. Moreover, transgene expression in some cases could interfere with normal plant growth and development due to an overdose of the functional proteins or dominant negative effect of non-functional products [3].

Transient gene expression provides a convenient alternative to stable transformation in analyzing gene function by virtue of its time and labor efficiency. It only takes one to several days to perform the assay in its entirety, which allows many constructs to be assayed in parallel within a short time and dramatically speeds up the pace of research. Routine transient assays include biolistic bombardment [4], protoplast transfection [5], and *Agrobacterium*-mediated transient assays [6], each with advantages and disadvantages depending on the research goals. The bombardment approach is simple and useful for a wide range of plant species. However, it depends strictly on expensive biolistic equipment and has an overall low transformation efficiency. Protoplast transfection also works for diverse plant species and could be used to investigate cell-autonomous regulation and responses in a quantitative and high-throughput way [7]. However, protoplast work requires substantial expertise, and has limited applicability in the studies where the cell wall or a tissue context is required.

*Agrobacterium*-mediated transient assays harness the natural capability of *Agrobacterium *to transfer foreign DNA into plant cells with an intact cell wall [8]. Among these assays, tobacco leaf infiltration [6] mediated by *Agrobacterium tumefaciens *is widely appreciated due to its easy operation and high transformation efficiency. However, when used to study genes from the model species Arabidopsis, tobacco as a heterologous system may not reflect the native activity or subcellular distribution of the protein in question [3,9]. Pilot efforts to explore an Arabidopsis equivalent of tobacco leaf infiltration have demonstrated low-frequency success with great variation [10-13]. Recently, a vacuum-infiltration procedure using young Arabidopsis seedling as *A. tumefaciens *target has been described [9], that broadly followed older protocols for tobacco and Arabidopsis seedling transformation [11,14]. On the other hand, cocultivation as a simplified alternative of infiltration has been successfully employed for transient gene expression in Arabidopsis seedlings using *Agrobacterium rhizogenes*. However, in this case the transient transformation was limited to the root epidermal cells [3]. Although *A. tumefaciens *cocultivation with Arabidopsis suspension cells has also been developed [15,16], the maintenance of Arabidopsis suspension cultured cells in healthy and aseptic conditions is a challenging and tedious task. In addition, Arabidopsis suspension cultured cells are dedifferentiated and do not provide normal tissue context.

In this study, we present an easy, efficient, and economical method for transient gene expression in young Arabidopsis seedlings based on *A. tumefaciens *cocultivation. This Fast Agro-mediated Seedling Transformation (FAST) method involves minimal hands-on manipulation and has no need for any specialized device, thus offering the potential of automation and high-throughput analysis of gene functions. As a proof of concept, we demonstrate the application of this method to the expression of various constructs in Arabidopsis seedlings with diverse genetic backgrounds for biochemical analysis, protein immunoblot, promoter tests, protein localization and protein-protein interaction studies. Furthermore, we show that this transient assay is also applicable to other important dicot and monocot species including tobacco, tomato, rice and switchgrass.

## Results

### Optimization of transient expression in the FAST assay

*A. tumefaciens *cocultivation method has been successfully adopted for the transformation of a wide range of plant species [17-21], which encouraged us to test whether *A. tumefaciens *cocultivation could also work for the transient transformation of Arabidopsis. We purposely chose young seedlings for a cocultivation test because (i) the generation of seedlings costs less time and space when compared with that of mature plants; (ii) the use of seedlings circumvents the problem that leaves of different ages from the same mature plant could have variable transformation efficiency.

**Figure 1:**
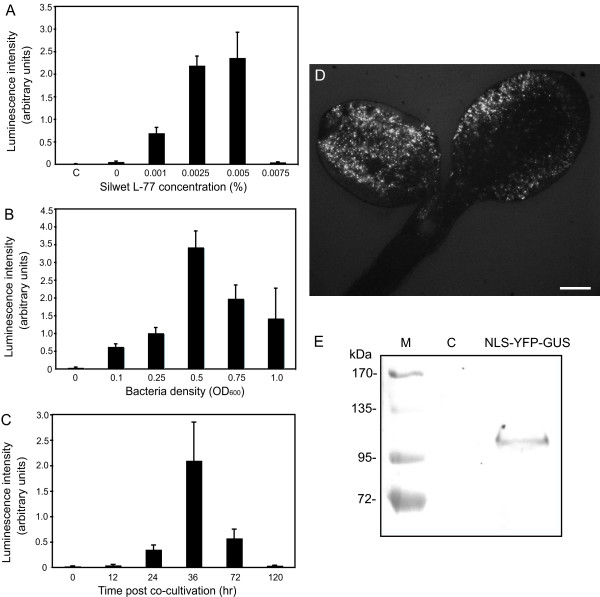
**Optimization of transient expression in the FAST assay**. A, Effect of Silwet L-77 concentration on transient expression. Transient expression efficiency was quantified by the average luminescence intensity of each Arabidopsis seedling expressing a *d35S::hRLuc *construct when Silwet L-77 of indicated concentration and bacteria of OD600 = 0.3 were included in the cocultivation medium. The assay was carried out after 40 hr cocultivation. The negative control indicated by letter "C" was performed using Agrobacteria cells carrying no binary vector. B, Effect of bacteria density on transient expression. Transient expression efficiency was quantified by the average luminescence intensity of each Arabidopsis seedling expressing a *d35S::hRLuc *construct when 0.005% Silwet L-77 and bacteria of indicated density were included in the cocultivation medium. The assay was carried out after 40 hr cocultivation. C, Time-course for transient expression. Transient expression efficiency was quantified by the average luminescence intensity of each Arabidopsis seedling at different time points during the cocultivation when 0.005% Silwet L-77 and bacteria of OD600 = 0.5 were used in the cocultivation medium. D, Expression of a *d35S::NLS-YFP-GUS *construct in a representative Arabidopsis seedling after 36 hr cocultivation when 0.005% Silwet L-77 and bacteria of OD600 = 0.5 were used in the cocultivation medium. Scale bar = 0.3 mm. E, Western blot analysis of NLS-YFP-GUS expression in 8 transformed (NLS-YFP-GUS) or untransformed (C) Arabidopsis seedlings after 36 hr cocultivation when 0.005% Silwet L-77 and bacteria of OD600 = 0.5 were used in the cocultivation medium.

When 4-day-old Arabidopsis Col-0 seedlings were cocultivated with *A. tumefaciens *GV3101 cells carrying the binary construct pVHK-NLS-YFP-GUS (Table 1), which allows the expression of a d35S promoter-driven nuclear targeted YFP-GUS fusion [22], only sporadic expression of this visible marker in cotyledon cells was detected under the fluorescence microscope (data not shown). Interestingly, we found that presence of the surfactant Silwet L-77 in the cocultivation medium dramatically boosted the transformation efficiency. Since Silwet L-77 concentration and agrobacterial density are positively correlated with transformation efficiency but are negatively correlated with viability of Arabidopsis seedlings, we first sought to determine the optimal Silwet L-77 concentration and agrobacteria density in this transient system. To this end, a d35S-driven codon-optimized *Renilla reniformis *luciferase (hRLuc) was utilized as an easily quantifiable marker [23] to monitor transient expression efficiency. When the final density of *A. tumefaciens *in the cocultivation medium was set to OD600 = 0.3 (approximately 3.6 × 10^8 ^cfu/mL), the highest hRLuc expression was detected with 0.005% (50 μL/L) Silwet L-77 in the medium, although lower concentrations of surfactant also yielded significant signal (Figure 1A). A higher Silwet L-77 concentration in the cocultivation medium led to accelerated necrosis of cotyledon cells as observed under the microscope (data not shown). When the Silwet L-77 concentration in the cocultivation medium was fixed at 0.005%, the highest hRLuc expression was achieved with a final *A. tumefaciens *density of OD600 = 0.5 (6 × 10^8 ^cfu/mL) (Figure 1B). Similarly, higher bacterial density in the medium elicited more severe necrosis of cotyledon cells (data not shown).

**Table 1:** Recombinant binary plasmids used in this study

Binary plasmid	Host vector	Resistance	Promoter	ORF	Reference
pPZP-hRLuc	pPZP222	Spectinomycin	d35S^a^	hRLuc	[23]
pVKH-NLS-YFP-GUS	pVKH18	Kanamycin	d35S	NLS-YFP-GUS	This study
px-cb	pFGC19	Kanamycin	d35S	Peroxisome-CFP	[25]
mt-yb	pFGC19	Kanamycin	d35S	Mitochondria-YFP	[25]
pFGC-YFP-1CCGT	pFGC19	Kanamycin	d35S	YFP-MYA1CCGT	[27]
pFGC-YFP-FABD2	pFGC19	Kanamycin	d35S	YFP-FABD2	This study
pVKH-YFP-At3g51660	pVKH18	Kanamycin	d35S	YFP-AT3g51660	This study
pVKH-At1g01170-YFP	pVKH18	Kanamycin	d35S	At1g01170-YFP	This study
pPZP-At2g47840-YFP	pPZP222	Spectinomycin	d35S	At2g47840-YFP	This study
pt-cb	pFGC19	Kanamycin	d35S	Plastid-CFP	[25]
ER-yk	pBIN20	Kanamycin	d35S	ER-YFP	[25]
ER-ck	pBIN20	Kanamycin	d35S	ER-CFP	[25]
G-yk	pBIN20	Kanamycin	d35S	Golgi-YFP	[25]
G-ck	pBIN20	Kanamycin	d35S	Golgi-CFP	[25]
mt-ck	pBIN20	Kanamycin	d35S	Mitochondria-CFP	[25]
px-ck	pBIN20	Kanamycin	d35S	Peroxisome-YFP	[25]
pt-yk	pBIN20	Kanamycin	d35S	Plastid-YFP	[25]
pVKH-YFP	pVKH18	Kanamycin	d35S	YFP	This study
pPZP-Cer	pPZP222	Spectinomycin	d35S	Cerulean	This study
pVKH-YFP-Cer	pVKH18	Kanamycin	d35S	YFP-Cerulean	This study
pBIN-YFP-HY5	pBIN20	Kanamycin	d35S	YFP-HY5	[23]
pPZP-Cer-HY5	pPZP222	Spectinomycin	d35S	Cerulean-HY5	This study
pVKH-YN-HY5	pVKH18	Kanamycin	d35S	YN-HY5	This study
pPZP-YC-HY5	pPZP222	Spectinomycin	d35S	YC-HY5	This study
pVKH-YFP-MYA1	pVKH18	Kanamycin	*MYA1pro*^b^	YFP-MYA1	This study
pVKH-GUS	pVKH18	Kanamycin	*Ubi-1*^c^	GUS	This study

^a ^d35S, double cauliflower mosaic virus 35S promoter.

^b ^*MYA1pro*, *MYA1 *native promoter.

^c ^*Ubi-1*, maize ubiquitin 1 promoter.

Note that 4 types of binary vectors with diverse genes have been tested.

Besides Silwet L-77 concentration and agrobacterial density, the time point of harvest and observation of the transformed seedlings is also an important parameter for this transient assay. Under the optimal Silwet concentration (0.005%) and *A. tumefaciens *density (OD600 = 0.5), the peak expression of hRLuc appeared after 36 hr cocultivation while further cocultivation led to increased necrosis of cotyledon cells until complete death of seedlings after 120 hr of cocultivation (Figure 1C). However, if the seedlings cocultivated for 36 hr were surface-sterilized with 1% bleach and transferred to a 0.25 × MS plate supplemented with 500 μg/mL carbenicillin and 1% sucrose, the transient expression in cotyledon cells could be sustained for as long as 10 days (see additional file 1). We also tested other factors in the cocultivation system such as the bacterial growth stage (from late log phase to early stationary phase), the seedling age (from 3-day-old to 5-day-old) and the pH value of cocultivation medium (from 5.7 to 6.5), all of which had only slight effects on the transient expression efficiency (data not shown). Interestingly, we found that older seedlings (more than 7-day-old) with emerging true leaves had a sharp decline in the transient expression efficiency, consistent with other recent reports [3,9,24]. The pre-induction of *Agrobacterium *virulence by acetosyringone, a step often conducted prior to *Agrobacterium *cocultivation/infiltration in other studies [3,17], was found to have little influence on transient expression and was thus omitted to save time. Based on these observations, a compact cocultivation procedure under optimized expression conditions (see Methods) was developed.

Under optimal cocultivation conditions, microscopic observation of the visible marker NLS-YFP-GUS revealed that its expression occurred among about 95% of the seedlings. The expression efficiency in individual cotyledons varied from 5% to nearly 100% but typically more than 50% of the cotyledon cells could be transformed (Figure 1D; also see additional file 2). This variability presumably stemmed from an uneven colonization of the plants by bacteria during the cocultivation period. Routinely, the NLS-YFP-GUS fluorescence was observed in the cotyledons, while the expression of this construct could also be detected in the petioles with lower frequency and in the hypocotyl occasionally (see additional file 2). However, no NLS-YFP-GUS expression could be visualized in roots (data not shown). The yield of NLS-YFP-GUS proteins in 8 transformed seedlings after 36 hr cocultivation was sufficient for western blot analysis, where a single band with molecular mass around 100 kDa was clearly recognized by polyclonal antibodies to GFP (Figure 1E).

### Expression of various constructs by the FAST assays

**Figure 2:**
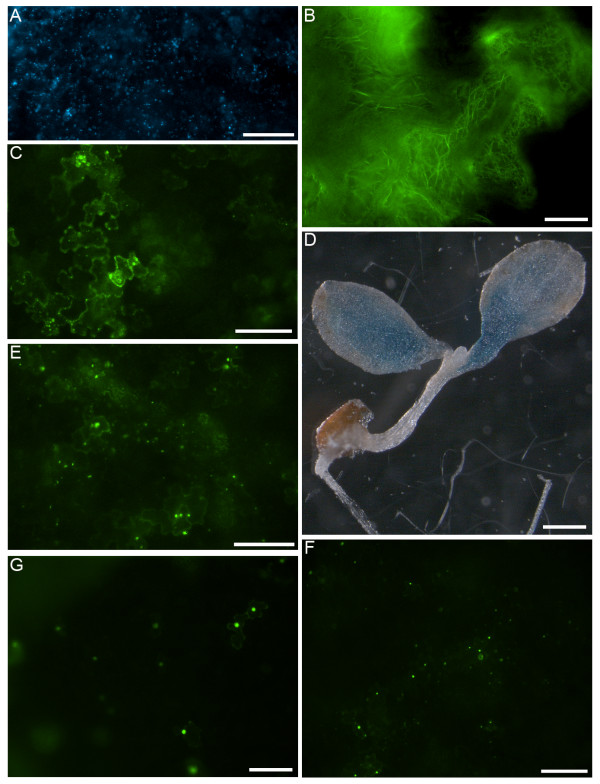
**Transient expression of various constructs in Arabidopsis by the FAST assays**. All cocultivations were carried out for 40 hr in the presence of 0.005% Silwet L-77 and bacteria of OD600 = 0.5. A, *d35S::Peroxisome-CFP *marker in wild-type seedling. Scale bar = 60 μm. B, *d35S::YFP-FABD2 *in wild-type seedling. Scale bar = 20 μm. C, *d35S::YFP-MYA1CCGT *in wild-type seedling. Scale bar = 60 μm. D, *ubi-1::GUS *in 33 wild-type seedling. Scale bar = 0.5 mm. E, *MYA1pro::YFP-MYA1 *in wild-type seedling. Scale bar = 60 μm. F, *MYA1pro::YFP-MYA1 *in *mya1 *mutant seedling. Scale bar = 60 μm. G, *d35S::NLS-YFP-GUS *in *eif3h *mutant seedling. Scale bar = 60 μm.

To ensure the reliability of the FAST system, the expression of a diverse array of constructs driven by various promoters in Arabidopsis seedlings with different genetic contexts was tested. We first employed this transient system to express d35S-driven organelle markers, namely Peroxisome-CFP and Mitochondria-YFP [25], in wild-type Arabidopsis seedlings. After 40 hr cocultivation, these two fluorescent proteins were visualized in numerous cotyledon cells (Figure 2A; data not shown) and were decorating different populations of motile punctate structures when co-expressed in the same cell (see additional file 5). By contrast, less than 5% of transformed cells demonstrated aberrant fluorescence distribution in the cytosol or nucleus in addition to proper organelle labeling. These abnormal cells all had exceptionally high levels of protein expression which could have been responsible for the partial mistargeting. Longer cocultivation (e.g. 72 hr) resulted in increased mistargeting of the organelle markers and cessation of the organelle movements (data not shown), suggesting that the extended cocultivation beyond the optimal time point (i.e., 36–40 hr) increased the stress to the cells and reduced the overall reliability of the assay. Besides compartment-targeted proteins, we also expressed a d35S-driven YFP-FABD2 construct, which clearly labeled the actin networks in the cytosol (Figure 2B) as described for its GFP version in a previous study [26]. Furthermore, we successfully expressed a d35S-driven YFP fusion of the truncated Arabidopsis myosin MYA1 protein including its C-terminal coiled-coil and globular tail domains, and found that this construct targeted to punctate structures in the cytosol (Figure 2C) as we had described earlier [27].

In addition to the d35S promoter, the activities of other promoters were also examined by FAST assays. The maize ubiquitin promoter *Ubi-1 *[28], which is frequently used as a strong promoter in monocots, was found to be also active in Arabidopsis (Figure 2D), as demonstrated by the expression of a *GUS *reporter gene under its control. Moreover, a YFP fusion of full-length Arabidopsis myosin MYA1 driven by its native promoter could also be properly expressed and bound to dynamic punctate structures in the cytosol (Figure 2E), consistent with its putative function in organelle trafficking [29].

Mutant Arabidopsis seedlings were also suitable for the FAST assay regardless of their seedling phenotypes. As an example, the YFP fusion of full-length MYA1 driven by its native promoter (Figure 2F) could be effectively expressed in *MYA1 *knockout mutant *mya1 *seedlings which did not show any obvious growth abnormality (E. Park unpublished data). In another example, the d35S-driven NLS-YFP-GUS construct could be readily expressed in *eif3h *mutant seedlings (Figure 2G), where the mutation itself caused strong defects in seedling growth [30]. These experiments suggested that this transient assay could be used for gene expression in Arabidopsis mutant lines that would die beyond the seedling stage due to a lethal mutation. Besides Col-0 ecotype seedlings, Ws ecotype seedlings could also be used to carry out this transient assay (see additional file 3). Similarly, *A. tumefaciens *strain GV3101 could be replaced by another strain, LBA4404, in the assay (see additional file 3).

### Subcellular localization studies using the FAST assays

Protein functions are largely restricted to defined locations in the cell [15,31]. Thus, identification of subcellular localization is one of the major uses of transient expression systems. We chose three uncharacterized Arabidopsis genes (i.e., At3g51660, At1g01170 and At2g47840) from the Arabidopsis subcellular database (SUBA, [32]) to illustrate this application. The intracellular distributions of all these gene products have not previously been determined cytologically, although mass spectrometry-based organelle proteomics has suggested their particular localizations. However, bioinformatic predictions listed in SUBA suggested ambiguous intracellular distributions for all of them (data not shown), which necessitates an experimental confirmation of their subcellular localizations with fluorescently tagged proteins.

**Figure 3:**
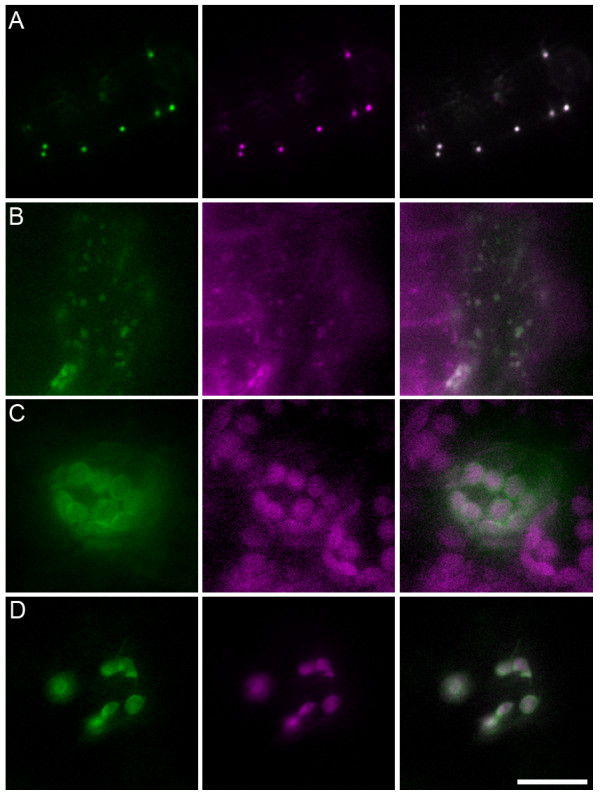
**Subcellular localization studies by the FAST assays**. A, YFP-At3g51660 (green) targeted to peroxisomes labeled by Peroxisome-CFP (Magenta) as indicated by the merged image. Arabidopsis seedlings were cocultivated simultaneously with agrobacteria cells carrying *d35S::YFP-At3g51660 *construct and those carrying *d35S::Peroxisome-CFP *marker. B, At1g01170-YFP (green) targeted to mitochondria labeled by Mitochondria-CFP (Magenta) as indicated by the merged image. Transgenic Arabidopsis seedlings expressing Mitochondria-CFP marker were cocultivated with agrobacteria cells carrying *d35S::At1g01170-YFP *construct. C, At2g47840-YFP (green) targeted to the envelope of chloroplasts labeled by autofluorescence (Magenta) as indicated by the merged image. Arabidopsis seedlings were cocultivated with agrobacteria cells carrying *d35S::At2g47840-YFP *construct. D, At2g47840-YFP (green) targeted to the envelope of plastids labeled by Plastid-CFP (Magenta) as indicated by the merged image. Arabidopsis seedlings were cocultivated with agrobacteria cells simultaneously carrying *d35S::At2g47840-YFP *and *d35S::Plastid-CFP *constructs. Scale bar = 15 μm.

The At3g51660 gene is annotated as encoding an Arabidopsis homolog of mammalian macrophage migration inhibitory factor (MIF). Several computer programs available in SUBA predicted its location to be in the cytosol, mitochondria, peroxisomes or the extracellular matrix while recent proteome analysis identified it as a peroxisomal protein [33]. In our transient assay, two *A. tumefaciens *strains carrying a *d35S::YFP-At3g51660 *construct and a *d35S::Peroxisome-CFP *marker [25], respectively, were included in the cocultivation with Arabidopsis seedlings, which led to the expression of YFP-At3g51660 proteins or Peroxisome-CFP markers in numerous cotyledon cells. Approximately 20% of the cells expressing the YFP-At3g51660 proteins were found to also express the Peroxisome-CFP markers. In all cases, the two fluorescent proteins colocalized in peroxisomes (Figure 3A), consistent with the proteomic data.

The At1g01170 gene is annotated as similar to ozone-induced protein AtOZI1, which is a stress-related protein accumulating in response to the production of reactive oxygen species [34]. Several prediction algorithms in SUBA suggested cytosol, plastid or extracellular location for the At1g01170 protein. Curiously, this protein was isolated with the mitochondrial proteome of suspension cell cultures [35]. Here we cocultivated *A. tumefaciens *cells carrying a *d35S::At1g01170-YFP *construct with transgenic Arabidopsis seedlings expressing a mitochondria-CFP marker [25]. The At1g01170-YFP hybrids were easily identified to target to mitochondria labeled by the specific CFP marker (Figure 3B), which agreed with previous proteomic data.

The At2g47840 gene is predicted as similar to AtTic20, which is a component of the protein-importing machinery at the inner envelope membrane of chloroplasts or plastids [36]. Although prediction programs in SUBA resulted in a range of different localizations including extracellular, mitochondrion, plastid or vacuole, the At2g47840 protein was identified as part of the chloroplast proteome [37]. When *A. tumefaciens *cells harboring a *d35S::At2g47840-YFP *construct were used for cocultivation with Arabidopsis seedlings, a ring-like fluorescence of At2g47840-YFP was detected in the transformed cotyledon cells, where the YFP signal was surrounding the chloroplast autofluorescence (Figure 3C), suggestive of the localization of At2g47840 protein to the chloroplast envelope. Moreover, when *A. tumefaciens *cells simultaneously carrying *d35S::At2g47840-YFP *and *d35S::plastid-CFP *constructs were used in the cocultivation, the YFP fluorescence was found to enclose the CFP-labeled plastid stroma (Figure 3D), again suggesting the localization of At2g47840 protein to the plastid envelope. These results not only validated earlier proteomic data, but also implied that both epidermal and mesophyll cells in the seedling could be readily transformed in the FAST assay.

**Figure 4:**
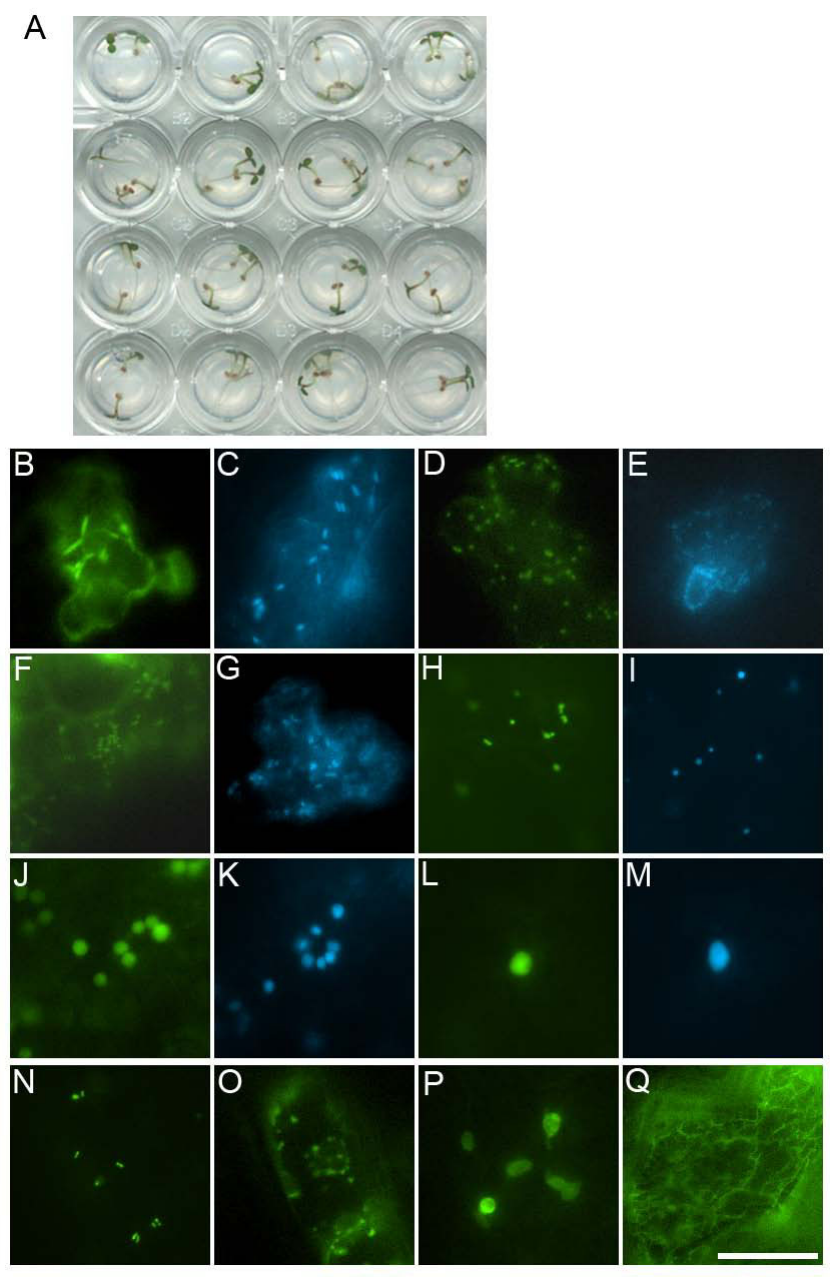
**The FAST assay in 96-well plate**. A, In a 4 × 4 grid of a 96-well plate, 2–3 Arabidopsis seedlings per well were soaked in 100 μL cocultivation medium containing 0.005% Silwet L-77 and bacteria of OD600 = 0.5. B-Q, Observation of protein expression after 40 hr cocultivation. B, ER-YFP; C, ER-CFP; D, Golgi-YFP; E, Golgi-CFP; F, Mitochondria-YFP; G, Mitochondria-CFP; H, Peroxisome-YFP; I, Peroxisome-CFP; J, Plastid-YFP; K, Plastid-CFP; L, YFP-HY5 labeling nucleus; M, Cerulean-HY5 labeling nucleus; N, YFP-At3g51660 labeling peroxisomes; O, At1g01170-YFP labeling mitochondria; P, At2g47840-YFP labeling plastids; Q, YFP-FABD2 labeling actin filaments. B-Q, Scale bar = 20 μm.

To test the feasibility of the FAST assay in 96-well plate which may benefit further high-throughput protein localization studies, sixteen different constructs were transiently expressed in the wells of a standard ELISA plate (96-well). These constructs included the YFP and CFP markers of ER, Golgi stacks, mitochondria, peroxisomes and plastids [25] as well as the YFP and Cerulean fusions of Arabidopsis transcription factor HY5 as nuclear markers [38]. In addition, the YFP fusions of At3g51660, At1g01170, At2g47840 and FABD2 constructs were also expressed. Each well contained 2–3 seedlings that were germinated directly in the well on 30 μL 0.25 × MS-agar. After 40 hr cocultivation, we detected the successful expression of every construct (Figure 4).

### Protein-protein interaction studies by the FAST assays

In addition to the proper subcellular localization, protein function is also dependent on or regulated by interactions with other proteins in the cell. Protein-protein interaction studies are ideally suited for transient assays since they allow tests of a larger number of construct combinations. Thus, we evaluated the efficiency of the FAST assay for the detection of *in vivo *protein-protein interactions by Förster resonance energy transfer (FRET) and bimolecular fluorescence complementation (BiFC) techniques. FRET relies on a non-radiative energy transfer from a fluorescent donor (e.g. Cerulean) to a fluorescent acceptor (e.g. YFP) when the donor and the acceptor are brought into close proximity by an interaction between their fusion partners [39]. BiFC is based on the reconstitution of a fluorescent protein (e.g. YFP) when its two halves (YN and YC) are brought into direct contact by stable interaction between their fusion partners [39]. In these assays, we took advantage of the high co-transformation efficiency when *A. tumefaciens *simultaneously carried two types of binary constructs [40]. As a proof of concept, we chose the Arabidopsis transcription factor HY5 [38] to test its homodimerization, which had been suggested by *in vivo *bioluminescence resonance energy transfer [41] and *in vitro *crystallographic experiments [42].

**Figure 5:**
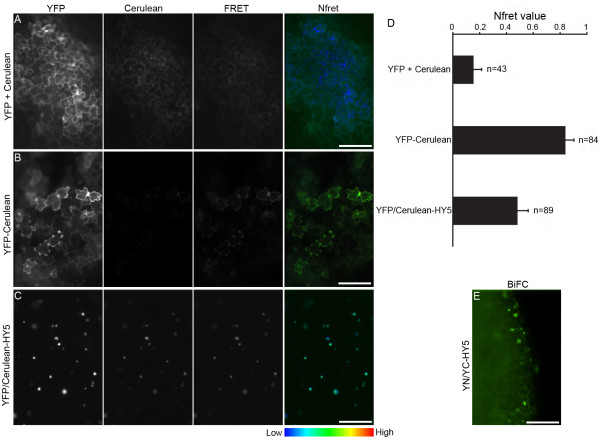
**FRET and BiFC evidence for HY5 homodimerization using the FAST assays**. Arabidopsis seedlings were cocultivated with agrobacteria cells simultaneously carrying two FRET or BiFC constructs except for (B). A, The negative FRET control, soluble YFP and Cerulean alone, showed a low Nfret (normalized FRET) value in a representative region of the cotyledon. Note blue color. Scale bar = 60 μm. B, The positive FRET control, a YFP-Cerulean fusion, showed a high Nfret value in a representative region of the cotyledon. Note green to yellow color. The remaining Cerulean signal was barely visible due to the intensive energy transfer from Cerulean to YFP. Scale bar = 60 μm. C, YFP-HY5 and Cerulean-HY5 showed a medium Nfret value in a representative region of the cotyledon. Note light blue to green color. Scale bar = 60 μm. D, Quantification of the average Nfret value in a large number of cells for each FRET combination indicated. E, Reconstituted YFP fluorescence in a BiFC combination of YN-HY5 and YC-HY5 in a representative region of the cotyledon. Scale bar = 60 μm.

In the FRET assay, we employed the normalized FRET (Nfret) calculation which removes spectral bleed-through and corrects for fluorophore expression level variation, and therefore is well-suited for widefield fluorescence microscopes [43]. For the negative FRET control, soluble YFP and Cerulean were found to be co-expressed in a large population of cotyledon cells after 40 hr cocultivation. As expected, these fluorescent proteins alone exhibited a low Nfret value (Figure 5A and 5D). By contrast, the positive FRET control of YFP-Cerulean fusion exhibited a high Nfret value (Figure 5B and 5D). In comparison, co-expressed YFP-HY5 and Cerulean-HY5 proteins showed a significant Nfret value (Figure 5C and 5D), which was markedly higher than that of the negative FRET control (Figure 5D), suggesting that HY5 proteins indeed homodimerize *in vivo*. The Nfret value of the HY5 homodimerization was lower than that of the positive FRET control (Figure 5D) as it resulted from a reversible protein-protein interaction rather than a permanent covalent attachment. Notably, although the expression levels of the fluorescent proteins in different cotyledon cells could be variable, we found that they did not affect the quantification of the Nfret value (see additional file 4; [43]), suggesting that the cell-to-cell variation of protein concentration does not prevent the FAST assay from producing reliable FRET data. The *in vivo *HY5 homodimerization detected by FRET could also be confirmed with the BiFC assay, where the reconstituted YFP fluorescence from YN-HY5 and YC-HY5 proteins was clearly visualized in the nuclei of many cotyledon cells after 40 hr cocultivation (Figure 5E).

### The FAST assays implemented in other plant species

**Figure 6:**
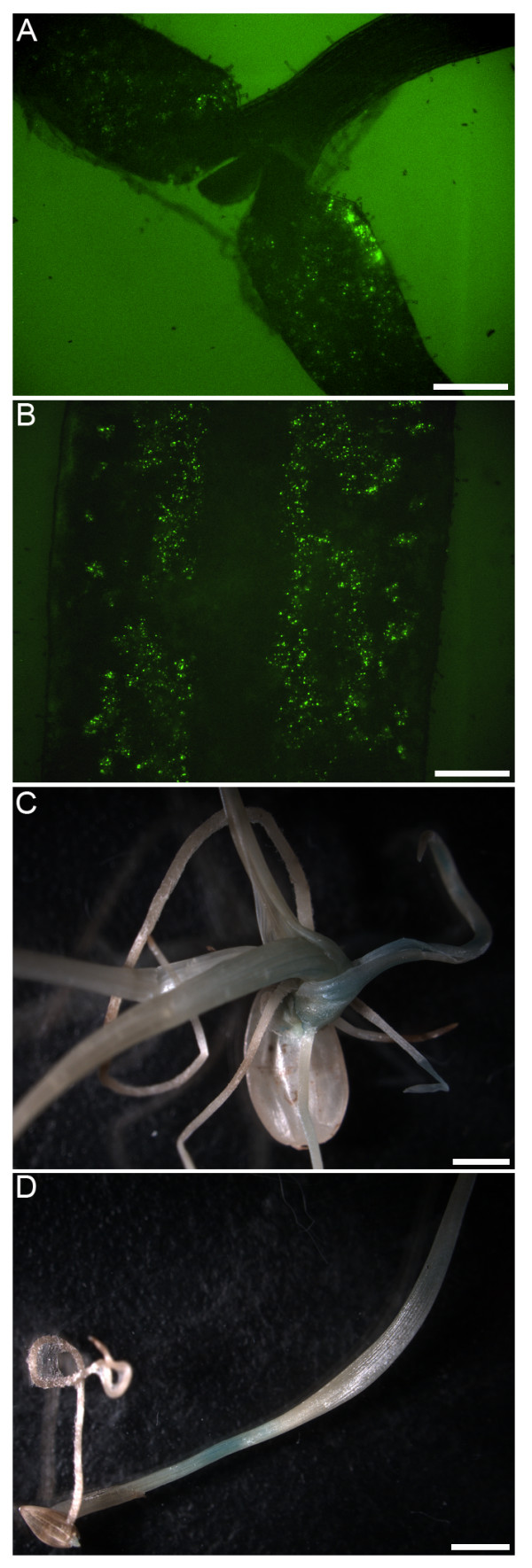
**The FAST assays in other plant species**. All cocultivations were carried out in the presence of 0.005% Silwet L-77 and bacteria of OD600 = 0.5. A, *d35S::NLS-YFP-GUS *expression in tobacco seedling after 40 hr of cocultivation. Scale bar = 0.5 mm. B, *d35S::NLS-YFP-GUS *expression in tomato seedling after 60 hr of cocultivation. Scale bar = 0.5 mm. C, *Ubi-1::GUS *expression in rice seedling after 6 days of cocultivation. Scale bar = 2 mm. D, *Ubi-1::GUS *expression in switchgrass seedling after 6 days of cocultivation. Scale bar = 2 mm.

We further tested the applicability of the FAST assay in other representative dicot species such as tobacco and tomato using the NLS-YFP-GUS construct as a visible marker. After 40–60 hr cocultivation, bright nuclei labeled by YFP fluorescence were found throughout the cotyledons of tobacco and tomato seedlings (Figure 6A and 6B). The total area where a detectable expression of NLS-YFP-GUS protein occurred after 40 hr cocultivation was relatively small, though 60 hr cocultivation could typically generate a broader area with elevated protein expression levels (Figure 6B). We also tested the suitability of this transient assay in key monocot species like rice and switchgrass using a *Ubi-1::GUS *construct as a reporter. Transient expression was pronounced in these organisms if the cocultivation time was extended to 6 days. Interestingly, GUS expression could be observed in different tissues of rice seedlings including shoot and root (Figure 6C), while in swichgrass seedlings only the shoot had detectable GUS expression (Figure 6D). The detection of GUS expression in intact switchgrass tissues in our assay system was in contrast to previous observations where GUS expression was limited to cut surfaces or wound sites [20]. Thus, the FAST protocol appeared to improve transformation efficiency even without deliberate optimization of cocultivation conditions.

## Discussion

Up to now, there was no available method that could achieve high transformation efficiency for transient gene expression in Arabidopsis with minimal manipulation. Pioneering efforts following the protocols similar to agro-infiltration of tobacco leaves led to rather limited success with great variability in Arabidopsis [10-13]. Very recently, an optimized *A. tumefaciens *vacuum infiltration protocol for young Arabidopsis seedlings had been described [9], which increased the overall success rate for obtaining transient expression in Arabidopsis. In this study, we explored a different strategy to transiently transform young Arabidopsis seedlings based on *A. tumefaciens *cocultivation, which we dubbed Fast Agro-mediated Seedling Transformation (FAST). Our data suggested that vacuum infiltration is not necessary as long as the surfactant Silwet L-77 is used in the FAST system (Figure 1; see additional file 2). Similarly, Silwet L-77 has previously been found to be critical in replacing laborious vacuum infiltration with the simple floral dip method in generating stably transformed Arabidopsis plants [2]. In addition, we found here that Silwet L-77, even at suboptimal concentration, could partially substitute for the stressful wounding step in catalyzing the *A. tumefaciens *transformation of recalcitrant monocot species such as switchgrass (Figure 6D; [20]). The lack of a wounding-requirement for the *Agrobacterium*-mediated transformation of plants is also consistent with earlier studies [44,45]. Thus, Silwet L-77 with a proper concentration is the key to achieve high transformation efficiency in this FAST system.

Following the FAST protocol, various constructs driven by different promoters were successfully expressed in young Arabidopsis seedlings with distinct genetic backgrounds including wild-type (Figure 1D; Figure 2A–E), mutant (Figure 2F and 2G) and transgenic seedlings (Figure 3B). The transient expression could occur in different organs of Arabidopsis seedlings including cotyledon, petiole and hypocotyl, and could occur in different cell types like epidermal and mesophyll cells (Figure 1D; see additional file 2). However, no transient expression could be detected in root (Figure 2D; data not shown). The tissue sensitivity of young Arabidopsis seedling to *A. tumefaciens *observed in this assay was in agreement with that described in a recent assay based on a vacuum infiltration method [9].

### The FAST assay has advantages over other expression systems *in planta*

Compared with stable transgenic assay, the FAST assay is extremely simple and rapid. The entire assay from sowing seeds to protein detection could be readily completed within one week, in contrast to two to three months generally required for obtaining transgenic plants. Moreover, this transient assay allows the expression of deleterious proteins which would disrupt the Arabidopsis growth and development when expressed in transgenic lines.

In comparison with existing transient assays available for Arabidopsis, the FAST assay also provides several particular advantages: (i) only routine techniques and reagents are used in this assay, which breaks the constraint of specialized device such as a particle gun and reduces the overall cost for the experiments; (ii) unlike particle bombardment and protoplast transfection where high-quality plasmid DNA has to be prepared each time, *A. tumefaciens *cells used in this assay can be stored indefinitely and can be repeatedly generated from the glycerol stock before use; (iii) this transient assay could achieve higher co-transformation efficiency for two constructs when they are simultaneously carried in the same agrobacteria cell; (iv) a small-scale assay has already produced enough proteins for downstream analysis (e.g. western blot), and the protein production in this assay could be easily scaled up for other applications (e.g. pull-down assay); (v) the use of 4-day-old Arabidopsis seedlings instead of mature plants allows for rapid screening with minimal manipulations, which could even be adapted for a 96-well plate format (Figure 4). Unlike detached leaves, mesophyll protoplasts, or suspension cultured cells, the seedling as an intact plant should provide a more physiological environment for gene functional study. Compared with the recently described seedling vacuum infiltration approach [9], the FAST assay offers the advantage that neither the pressurizing device nor the supporting grid and fewer manual handling steps are required in the process.

### The FAST assay is efficient in protein localization and protein-protein interaction studies

Although the variation of transformation efficiency from seedling to seedling is substantial in our transient system (see additional file 2) presumably due to an uneven distribution of agrobacteria during cocultivation, this has been significantly compensated by the advantage that the number of seedlings screenable in an assay could be very high. Thus, an overall constant output can be obtained. For a small-scale experiment using 30 seedlings, thousands of cells were readily transformed which could be screened under microscope within two hours and which should be more than enough to generate reliable protein localization or protein-protein interaction data.

The variation of gene expression from cell to cell is generally the major concern for a transient expression system. In the FAST assay, although we also detected various levels of protein expression in different cotyledon cells (Figure 2; see additional file 4), we have not noticed any alteration of protein targeting stemming from this variability except a partial mistargeting in a small percentage of cells (around 5%). This partial mistargeting likely was due to the extremely high concentration of introduced proteins in these cells, which may have led to the overflow of the compartment and a subsequent diffuse staining or aggregation of the proteins in the cytoplasm or nucleus. However, it is quite easy to gain thousands of transformed cells in a small-scale assay using 30 seedlings. Since the transient expression in each cell can be considered an independent event, the average distribution pattern of a given construct in a large population of cells should be considered typical. In protein-protein interaction studies, the variation of protein levels in different cells had negligible impact on FRET quantification when a proper algorithm such as Nfret [43] was used (see additional file 4).

Another possible concern is that high bacteria density and surfactant concentration may be stressful to the seedling cells and may generate secondary effects on protein localization. However, it has been suggested that the *Agrobacterium *infection generally would not change intracellular protein localization [46]. Indeed, the overexpressed proteins in this study could faithfully target to cytoplasm, nucleus, organelles or specific cytoplasmic structures (e.g. actin filaments) in the vast majority of transformed cells at the appropriate observation time (i.e., after 36–40 hr cocultivation). Moreover, the vigorous movements of peroxisomes and mitochondria labeled by fluorescent organelle markers (see additional file 5) in the majority of transformed cells also is a reflection of their good physiological conditions, suggesting that this pathogen-associated transient assay was not very disturbing to normal cell physiology. Nevertheless, lower bacteria density and surfactant concentration in the cocultivation medium could create a milder environment which would be closer to the physiological conditions. In fact, we noticed that an *A. tumefaciens *density of OD600 = 0.1 (1.2 × 10^8 ^cfu/mL) and Silwet L-77 concentration as low as 0.002% could still generate large numbers of transformed cells and could on average reduce the overexpression level in the cell (data not shown).

To co-express two proteins in the same plant cell is the key for protein localization and protein-protein interaction studies. Here, we preferred to cocultivate the Arabidopsis seedlings with *A. tumefaciens *cells simultaneously carrying two compatible binary plasmids, a strategy modeled after the dual-binary T-DNA system [40]. Since each binary plasmid contains the expression cassette for one protein, a theoretical 100% co-transformation efficiency could be guaranteed when a given plant cell is transformed by an *A. tumefaciens *cell. However, in practice, a co-transformation rate of approximately 70% was observed (data not shown). The reason behind this phenomenon is obscure but may be related to the nature of the different binary vectors and the encoded genes, as these two factors have previously been suggested to influence the transformation efficiency and the level of transient gene expression [3,11,15]. In protein-protein interaction studies, the high co-transformation efficiency enabled the co-expression of a protein pair (e.g. YFP-HY5 and Cerulean-HY5) in numerous cotyledon cells, which facilitated an easy acquisition of fluorescence data from a large number of cells for statistically significant FRET quantification and an easy detection of reconstituted YFP fluorescence in BiFC experiments.

### Limitations of the FAST assay

While the FAST assay offers a number of advantages, it also comes with a few limitations that may reduce its usefulness in certain situations. For example, while we have observed high transformation rates in cotyledons, we did not detect any transgene expression in other tissues such as roots or true leaves, which could be relevant for interactions with endogenous proteins that are not expressed in cotyledons. Also, since cocultivation with *A. tumefaciens *could potentially induce host defenses as recently reported for *A. tumefaciens *infiltration [47], the FAST assay may not be appropriate for functional analysis of genes involved in plant defense response. However, this may not always be the case [12] and should be tested on a case by case basis.

## Conclusion

We have demonstrated here that the FAST assay is efficient for the rapid examination of biochemical activity, subcellular localization and interacting partners of a gene product. It is also useful for a quick test of the expression of binary constructs before undertaking stable transformation. Moreover, this assay provides a convenient tool for foreign protein overexpression *in planta*, especially in Arabidopsis. In principle, we envision that this transient assay should also be effective in producing hairpin or antisense RNA for RNA silencing research in plants. Notably, due to the minimal manual handling and high benefit-to-cost ratio in terms of time, labor and money, this transient assay is ideal for a further automated and high-throughput survey of plant gene functions.

## Methods

### Plasmid construction

Routine molecular cloning techniques were followed to construct the various binary constructs (Table 1), which were then transformed into *A. tumefaciens *cells (strain GV3101::pMP90; [48]) by electroporation. In some cases (e.g. for protein-protein interaction studies), two binary constructs with different antibiotic resistances (i.e., kanamycin and spectinomycin, respectively) were simultaneously transformed into the same *A. tumefaciens *cell by co-electroporation, which was previously described as dual-binary T-DNA strategy [40]. Single or double transformants of *A. tumefaciens *were selected on LB medium with corresponding antibiotics.

### Seedling growth

*Arabidopsis thaliana *(Col-0) seeds were surface sterilized in 30% household bleach (1.5% sodium hypochlorite at final concentration) and 0.1% Triton X100 for 10 min before they were sown on 0.25 × Murashige-Skoog (MS)-phytoblend (Caisson Laboratories, ) plate (pH 6.0) containing 1% sucrose. After stratification at 4°C for 24 hr, Arabidopsis seeds were germinated in a cycle of 16 hr light/22°C followed by 8 hr dark/18°C. Tobacco (*Nicotiana benthamiana*) seeds were sterilized in 10% bleach (0.5% sodium hypochlorite) and 0.1% Tween 20 for 10 min and 70% ethanol for 1 min, while tomato (*Solanum lycopersicum*) seeds were sterilized in 70% ethanol for 3 min before they were directly germinated under the same condition as Arabidopsis seeds except without cold stratification. Switchgrass (*Panicum virgatum*) seeds and dehulled rice (*Oryza sativa*) seeds, without sterilization, were directly germinated under the same condition as dicot seeds on germination paper wetted with sterilized deionized water.

### Fast agro-mediated seedling transformation

The day before cocultivation, liquid cultures of *A. tumefaciens *were inoculated from colonies on agar plates or frozen glycerol stock. After growth at 28°C in 2 mL LB medium with appropriate antibiotics (25 μg/mL streptomycin plus 50 μg/mL kanamycin or 100 μg/mL spectinomycin, or both) for 18–24 hr, 1.6 mL saturated culture was diluted the next day into 10 mL fresh YEB medium (5 g/L beef extract, 1 g/L yeast extract, 5 g/L peptone, 5 g/L sucrose, 0.5 g/L MgCl_2_) to OD600 = 0.3 and was grown until the OD_600 _reached more than 1.5. Bacteria cells were harvested by centrifugation at 6,000 g for 5 min and washed once with 10 mL washing solution containing 10 mM MgCl_2 _and 100 μM acetosyringone. After centrifugation at 6,000 g for another 5 min, the pellet of bacteria cells was resuspended in 1 mL washing solution. We also tested a simplified protocol where *A. tumefaciens *cells were directly scraped from agar plates and resuspended into wash solution at a final OD600 = 0.3. This simplified procedure resulted in similar transformation efficiency as the protocol described above.

In a clean Petri dish (100 × 20 mm), 30–40 4-day-old Arabidopsis (or tobacco) seedlings, 10–15 4-day-old tomato seedlings or 5-day-old rice (or swichgrass) seedlings were soaked with 20 mL cocultivation medium containing 0.25 × MS (pH 6.0, Caisson Laboratories), 1% sucrose, 100 μM acetosyringone, 0.005% (v/v; i.e. 50 μL/L) Silwet L-77 and *A. tumefaciens *cells at final density of OD600 = 0.5 (6 × 10^8 ^cfu/mL). Cocultivation was carried out in darkness at the same temperature as seedling growth for 36–40 hr for Arabidopsis seedlings, 36–60 hr for tobacco (or tomato) seedlings or 6 days for rice (or switchgrass) seedlings before microscopic observation or other analysis was performed. For cocultivation assay in 96-well plate, 2–3 4-day-old Arabidopsis seedlings geminated directly on 30 μL MS-agar (0.8%) plus 1% sucrose in each well were cocultivated with *A. tumefaciens *cells in 100 μL cocultivation medium.

### Luciferase assay in seedlings

For every combination of cocultivation conditions, six independent experiments were carried out to express a *d35S::hRLuc *construct, each using 5 Arabidopsis seedlings. Prior to sampling, these 5 seedlings were surface-sterilized with 1% bleach (0.05% sodium hypochlorite) for 10 min to remove epiphytic bacteria and then were washed with sterile distilled water three times. The seedlings were placed in a microcentrifuge tube and covered with 100 μL 2 μM coelenterazine solution. The total luminescence of these five Arabidopsis seedlings was immediately measured using a TD-20/20 luminometer (Turner Designs, ) with sensitivity, duration of measurement and delay time setting as 35%, 10 sec and 10 sec, respectively [23].

### GUS histochemical assay

Plant seedlings with GUS expression were incubated with prechilled 90% acetone at room temperature for 20 min. After extensive washing, seedlings were immersed into fresh staining solution containing 1 mM X-Gluc in 50 mM Na_3_PO_4 _(pH 7.2), 0.2% Triton X-100, 0.2 mM K_3_Fe(CN)_6 _and 0.2 mM K_4_Fe(CN)_6_. Vacuum treatment was applied to switchgrass and rice seedlings to facilitate the penetration of the staining solution. The staining was carried out at 37°C in the dark for 12 hr for Arabidopsis seedlings or 36 hr for switchgrass and rice seedlings, after which chlorophyll was removed from seedlings by extensive destaining with 70% ethanol. The GUS-stained seedlings were imaged under a Leica MZ 16 FA fluorescence stereomicroscope (Meyer instruments, ).

### Total protein extraction and western blot

After microscopic analysis, 8 transformed or untransformed seedlings were placed in a microcentrifuge tube and homogenized by grinding with a micropestle in the presence of 20 μL extraction buffer containing 50 mM Tris-HCl (pH 7.5), 150 mM NaCl, 10 mM DTT, 2.5 mM EDTA, 0.1% Triton X-100, 1 mM PMSF and 1 × Complete protease 24 inhibitors (Roche, ). 5 μL 5 × SDS loading buffer was added and the resultant mixture was boiled for 10 min. The supernatant after a centrifugation at 3,000 g for 5 min was loaded for SDS-PAGE analysis. Western analysis was performed as described earlier [49] with polyclonal rabbit anti-GFP antibody (Invitrogen, ) as primary antibody.

### Live cell imaging and FRET measurement

Transformed plant seedlings expressing fluorescent proteins were observed using an Axiovert 200 M inverted microscope (Zeiss, ) equipped with filters for YFP and CFP/Cerulean fluorescence (Chroma , filter set 52017). A 20 × objective was first used to identify the transformed cells and set up the focus plane before 2.5 × objective or 63 × (1.4 NA) plan-apo oil immersion objective was used, respectively, to obtain an overview of seedling or to examine a transformed region at a higher resolution. Images were captured with a digital camera (Hamamatsu Orca-ER, ) controlled by OpenLab software (Improvision, ). FRET measurements were performed as previously described [27]. Briefly, images were acquired sequentially through Cerulean, FRET and YFP filter channels. Normalized FRET (Nfret) with subtracted spectral bleed-through and correction of cellular fluorophore concentration was calculated by OpenLab software (Improvision) using the Nfret algorithm [43]. Areas with substantial image signals in all three channels were manually selected and the mean Nfret values for those areas were determined using OpenLab software (Improvision).

## Competing interests

The authors declare that they have no competing interests.

## Authors' contributions

JFL and AN designed the experiments. JFL, EP and AVA performed the experiments. JFL and AN wrote the manuscript. All authors read and approved the final manuscript.

## Supplementary Material

Additional File 1 **Transient expression in a transformed Arabidopsis seedling could last for 10 days**. After 36 hr cocultivation, Arabidopsis seedling expressing a *d35S::NLS-YFP-GUS *construct (A) was surface-sterilized with 1% bleach for 10 min, washed with sterile water three times, and transferred to 0.25 × MS plate supplemented with 500 μg/mL carbenicillin and 1% sucrose. Transient expression in the cotyledon cells of the same seedling could still be detected 5 days (B) or 10 days (C) post the cocultivation. Letters "Co" and "L" denote cotyledon and leaf, respectively. Scale bar = 0.5 mm. Click here for file

Additional File 2 **Variation of transformation efficiency in the FAST assay under optimal cocultivation conditions**. Transient expression of a *d35S::NLS-YFP-GUS *construct in different Arabidopsis seedlings (A-H) after 36 hr cocultivation when 0.005% Silwet L-77 and bacteria of OD600 = 0.5 were used in the cocultivation medium. Scale bar = 0.5 mm. Click here for file

Additional File 3 **Arabidopsis ecotype Col-0 seedlings and *A. tumefaciens *strain GV3101 in the FAST assay could be replaced by ecotype Ws seedlings and *A. tumefaciens *strain LBA4404, respectively**. A, Arabidopsis ecotype Ws seedlings were cocultivated with *A. tumefaciens *strain GV3101 carrying *d35S::Peroxisome-CFP *marker. B, Arabidopsis ecotype Ws seedlings were cocultivated with *A. tumefaciens *strain GV3101 carrying *d35S::Plastid-CFP *marker. C, Arabidopsis ecotype Col-0 seedlings were cocultivated 37 with *A. tumefaciens *strain LBA4404 carrying *d35S::Peroxisome-CFP *marker. D, Arabidopsis ecotype Col-0 seedlings were cocultivated with *A. tumefaciens *strain LBA4404 carrying *d35S::Plastid-CFP *marker. Scale bar = 60 μm. Click here for file

Additional File 4 **Variation of transient expression levels in different cotyledon cells does not affect FRET quantification when Nfret algorithm was used**. The FRET quantification by Nfret algorithm for 43 cells expressing YFP-Cerulean fusion showed that the Nfret value of these cells was almost constant irrespective of the variable cellular YFP concentration. Note that the regression line runs parallel to the x-axis. Click here for file

Additional File 5 **Organelle dynamics in a transformed cotyledon cell**. Peroxisomes and mitochondria in the cell were respectively labeled by Peroxisome-CFP (red) and Mitochondria-YFP (green) constructs expressed by the FAST assay. Cocultivation was carried out for 40 hr in the presence of 0.005% Silwet L-77 and bacteria of OD600 = 0.5. 60 serial images of the cell were taken every second for a duration of 1 min. Click here for file

## References

[B1] Edwards D, Batley J (2004). Plant bioinformatics: from genome to phenome. Trends Biotechnol.

[B2] Clough SJ, Bent AF (1998). Floral dip: a simplified method for *Agrobacterium*-mediated transformation of *Arabidopsis thaliana*. Plant J.

[B3] Campanoni P, Sutter JU, Davis CS, Littlejohn GR, Blatt MR (2007). A generalized method for transfecting root epidermis uncovers endosomal dynamics in Arabidopsis root hairs. Plant J.

[B4] Christou P (1995). Strategies for variety-independent genetic transformation of important cereals, legumes and woody species utilizing particle bombardment. Euphytica.

[B5] Sheen J (2001). Signal transduction in maize and Arabidopsis mesophyll protoplasts. Plant Physiol.

[B6] Yang Y, Li R, Qi M (2000). In vivo analysis of plant promoters and transcription factors by agroinfiltration of tobacco leaves. Plant J.

[B7] Yoo SD, Cho YH, Sheen J (2007). Arabidopsis mesophyll protoplasts: a versatile cell system for transient gene expression analysis. Nat Protoc.

[B8] Gelvin SB (2003). *Agrobacterium*-mediated plant transformation: the biology behind the gene-jockeying tool. Microbiol Mol Biol Rev.

[B9] Marion J, Bach L, Bellec Y, Meyer C, Gissot L, Faure JD (2008). Systematic analysis of protein subcellular localization and interaction using high-throughput transient transformation of Arabidopsis seedlings. Plant J.

[B10] Rakousky S, Kocabek T, Vincenciova R, Ondrej M (1997). Transient β-glucuronidase activity after infiltration of *Arabidopsis thaliana *by *Agrobacterium tumefaciens*. Biol Plant.

[B11] McIntosh KB, Hulm JL, Young LW, Bonham-Smith PC (2004). A rapid *Agrobacterium*-mediated *Arabidopsis thaliana *transient assay system. Plant Mol Biol Rep.

[B12] Wroblewski T, Tomczak A, Michelmore R (2005). Optimization of *Agrobacterium*-mediated transient assays of gene expression in lettuce, tomato and Arabidopsis. Plant Biotechnol J.

[B13] Lee MW, Yang Y (2006). Transient expression assay by agroinfiltration of leaves. Methods Mol Biol.

[B14] Rossi L, Escudero J, Hohn B, Tinland B (1993). Efficient and sensitive assay for T-DNA-dependent transient gene expression. Plant Mol Biol Rep.

[B15] Koroleva OA, Tomlinson ML, Leader D, Shaw P, Doonan JH (2005). High-throughput protein localization in Arabidopsis using *Agrobacterium*-mediated transient expression of GFP-ORF fusions. Plant J.

[B16] Berger B, Stracke R, Yatusevich R, Weisshaar B, Flügge UI, Gigolashvili T (2007). A simplified method for the analysis of transcription factor-promoter interactions that allows high-throughput data generation. Plant J.

[B17] Li XQ, Liu CN, Ritchie SW, Peng JY, Gelvin SB, Hodges TK (1992). Factors influencing *Agrobacterium*-mediated transient expression of *gusA *in rice. Plant Mol Biol.

[B18] Hadfi K, Batschauer A (1994). *Agrobacterium*-mediated transformation of white mustard (*Sinapis alba *L.) and regeneration of transgenic plants. Plant Cell Rep.

[B19] Zhou J, Wei Z, Xu Z, Liu S, Luo P (1996). *Agrobacterium tumefaciens *mediated transformation of *Orychophragmus violaceus *cotyledon and regeneration of transgenic plants. Chin J Biotechnol.

[B20] Somleva MN, Tomaszewski Z, Conger BV (2002). *Agrobacterium*-mediated genetic transformation of switchgrass. Crop Sci.

[B21] Grant JE, Cooper PA, Dale TM (2004). Transgenic *Pinus radiate *from *Agrobacterium tumefaciens*-mediated transformation of cotyledons. Plant Cell Rep.

[B22] Grebenok RJ, Pierson E, Lambert GM, Gong FC, Afonso CL, Haldeman-Cahill R, Carrington JC, Galbraith DW (1997). Green-fluorescent protein fusions for efficient characterization of nuclear targeting. Plant J.

[B23] Subramanian C, Woo J, Cai X, Xu X, Servick S, Johnson CH, Nebenführ A, von Arnim AG (2006). A suite of tools and application notes for *in vivo *protein interaction assays using bioluminescence resonance energy transfer (BRET). Plant J.

[B24] McDowell JM, Williams SG, Funderburg NT, Eulgem T, Dangl JL (2005). Genetic analysis of developmentally regulated resistance to downy mildew (*Hyaloperonospora parasitica*) in *Arabidopsis thaliana*. Mol Plant Microbe Interact.

[B25] Nelson BK, Cai X, Nebenführ A (2007). A multicolored set of in vivo organelle markers for co-localization studies in Arabidopsis and other plants. Plant J.

[B26] Voigt B, Timmers AC, Samaj J, Muller J, Baluska F, Menzel D (2005). GFP-FABD2 fusion construct allows *in vivo *visualization of the dynamic actin cytoskeleton in all cells of Arabidopsis seedlings. Eur J Cell Biol.

[B27] Li JF, Nebenführ A (2008). Inter-dependence of dimerization and organelle binding in myosin XI. Plant J.

[B28] Christensen AH, Quail PH (1996). Ubiquitin promoter-based vectors for high-level expression of selectable and/or screenable marker genes in monocotyledonous plants. Transgenic Res.

[B29] Li JF, Nebenführ A (2008). The tail that wags the dog: the globular tail domain defines the function of myosin V/XI. Traffic.

[B30] Kim TH, Kim BH, Yahalom A, Chamovitz DA, von Arnim AG (2004). Translational regulation via 5' mRNA leader sequences revealed by mutational analysis of the Arabidopsis translation initiation factor subunit eIF3h. Plant Cell.

[B31] Komatsu S, Konishi H, Hashimoto M (2007). The proteomics of plant cell membranes. J Exp Bot.

[B32] Heazlewood JL, Verboom RE, Tonti-Filippini J, Small I, Millar AH (2007). SUBA: the Arabidopsis subcellular database. Nuleic Acids Res.

[B33] Reumann S, Babujee L, Ma C, Wienkoop S, Siemsen T, Antonicelli GE, Rasche N, Luder F, Weckwerth W, Jahn O (2007). Proteome analysis of Arabidopsis leaf peroxisomes reveals novel targeting peptides, metabolic pathways, and defense mechanisms. Plant Cell.

[B34] Sharma YK, Davis KR (1995). Isolation of a novel Arabidopsis ozone-induced cDNA by differential display. Plant Mol Biol.

[B35] Brugiere S, Kowalski S, Ferro M, Seigneurin-Berny D, Miras S, Salvi D, Ravanel S, d'Herin P, Garin J, Bourguignon J, Joyard J, Rolland N (2004). The hydrophobic proteome of mitochondrial membranes from Arabidopsis cell suspensions. Phytochemistry.

[B36] Chen X, Smith MD, Fitzpatrick L, Schnell DJ (2002). *In vivo *analysis of the role of atTic20 in protein import into chloroplasts. Plant Cell.

[B37] Kleffmann T, Russenberger D, von Zychlinski A, Christopher W, Sjolander K, Gruissem W, Baginsky S (2004). The *Arabidopsis thaliana *chloroplast proteome reveals pathway abundance and novel protein functions. Curr Biol.

[B38] Oyama T, Shimura Y, Okada K (1997). The Arabidopsis *HY5 *gene encodes a bZIP protein that regulates stimulus-induced development of root and hypocotyl. Genes Dev.

[B39] Lalonde S, Ehrhardt DW, Loque D, Chen J, Rhee SY, Frommer WB (2008). Molecular and cellular approaches for the detection of protein-protein interactions: latest techniques and current limitations. Plant J.

[B40] Afolabi AS, Worland B, Snape JW, Vain P (2004). A large scale study of rice plants transformed with different T-DNAs provides new insights into locus composition and T-DNA linkage configurations. Theor Appl Genet.

[B41] Subramanian C, Xu Y, Johnson CH, von Arnim AG (2004). *In vivo *detection of protein-protein interaction in plant cells using BRET. Methods Mol Biol.

[B42] Yoon MK, Kim HM, Choi G, Lee JO, Choi BS (2007). Structural basis for the conformational integrity of the *Arabidopsis thaliana *HY5 leucine zipper homodimer. J Biol Chem.

[B43] Xia Z, Liu Y (2001). Reliable and global measurement of fluorescence resonance energy transfer using fluorescence microscopes. Biophys J.

[B44] Escudero J, Hohn B (1997). Transfer and integration of T-DNA without cell injury in the host plants. Plant Cell.

[B45] Brencic A, Angert ER, Winans SC (2005). Unwounded plants elicit Agrobacterium vir gene induction and T-DNA transfer: transformed plant cells produce opines yet are tumor free. Mol Microbiol.

[B46] Goodin MM, Chakrabarty R, Banerjee R, Yelton S, DeBolt S (2007). New gateways to discovery. Plant Physiol.

[B47] Pruss GJ, Nester EW, Vance V (2008). Infiltration with *Agrobacterium tumefaciens *induces host defense and development-dependent responses in the infiltrated zone. Mol Plant Microbe Interact.

[B48] Koncz C, Schell J (1986). The promoter of TL-DNA gene 5 controls the tissue-specific expression of chimeric genes carried by a novel type of *Agrobacterium *binary vector. Mol Gen Genet.

[B49] Li JF, Qu LH, Li N (2005). Tyr152 plays a central role in the catalysis of 1-aminocyclopropane-1-carboxylate synthase. J Exp Bot.

