# Supplementary figures and images for: The FAST technique: a simplified Agrobacterium-based transformation method for transient gene expression analysis in seedlings of Arabidopsis and other plant species
Source: Plant Methods. 2009 May 20;5:6. doi: 10.1186/1746-4811-5-6 (PMC2693113; doi:10.1186/1746-4811-5-6)

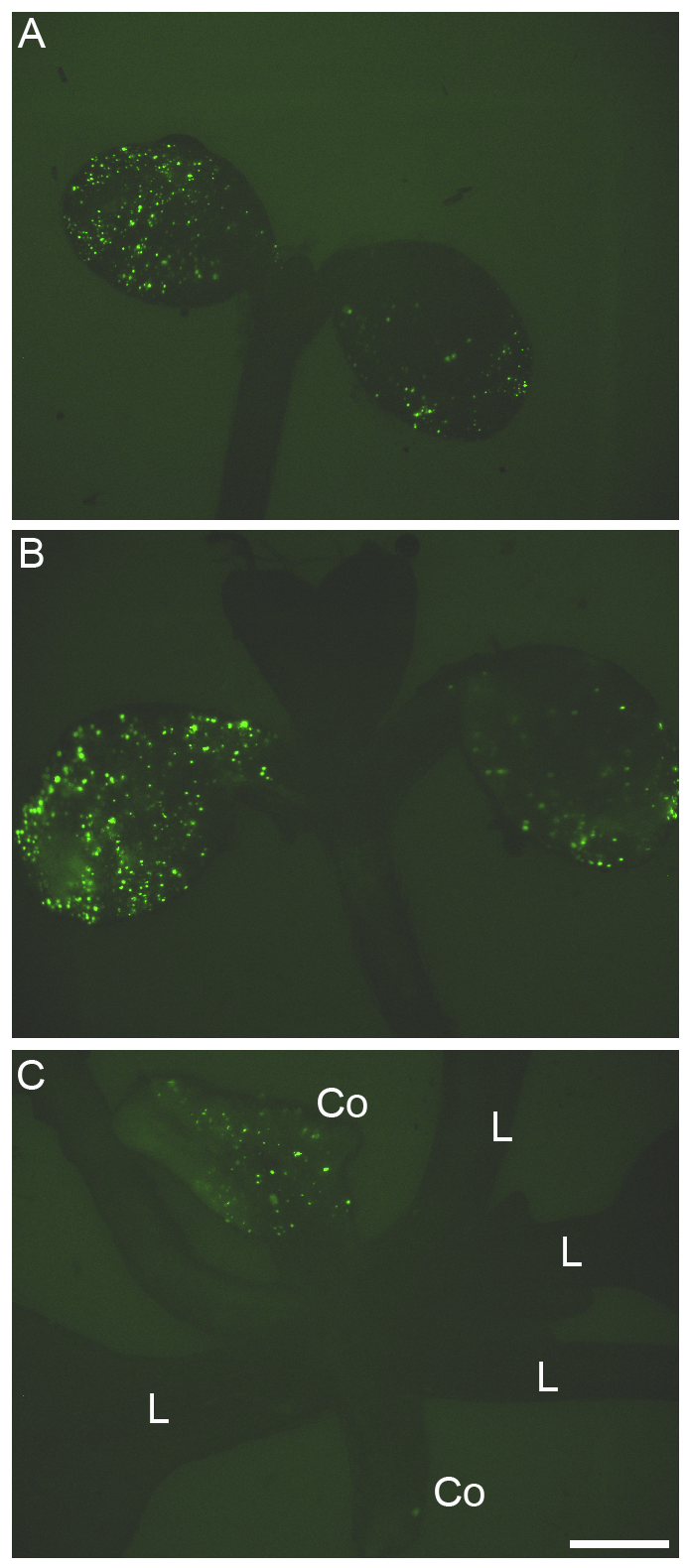

Supplement: Additional File 1 — Transient expression in a transformed Arabidopsis seedling could last for 10 days. After 36 hr cocultivation, Arabidopsis seedling expressing a d35S::NLS-YFP-GUS construct (A) was surface-sterilized with 1% bleach for 10 min, washed with sterile water three times, and transferred to 0.25 × MS plate supplemented with 500 μg/mL carbenicillin and 1% sucrose. Transient expression in the cotyledon cells of the same seedling could still be detected 5 days (B) or 10 days (C) post the cocultivation. Letters "Co" and "L" denote cotyledon and leaf, respectively. Scale bar = 0.5 mm. [file 1746-4811-5-6-S1.tiff]

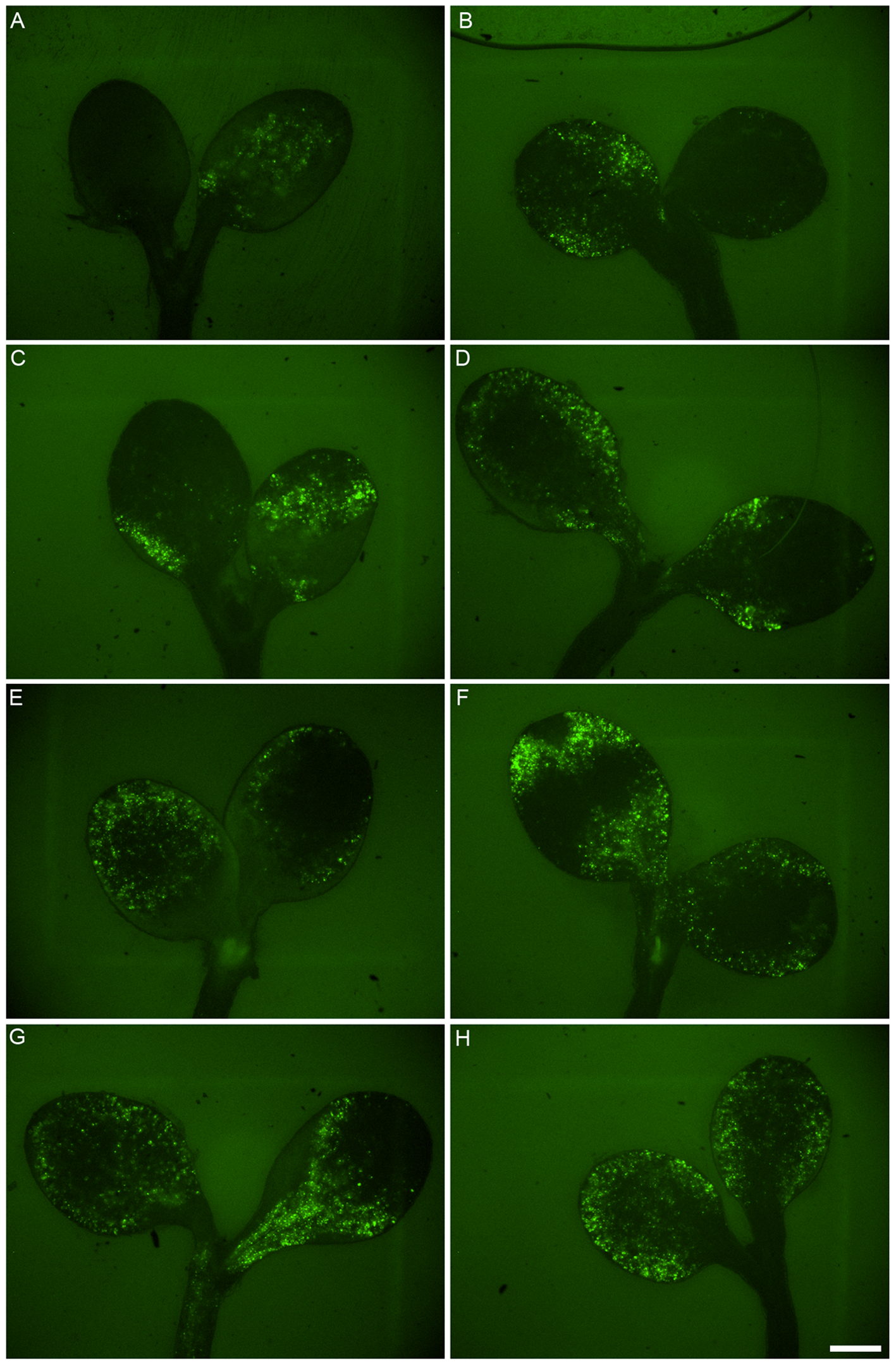

Supplement: Additional File 2 — Variation of transformation efficiency in the FAST assay under optimal cocultivation conditions. Transient expression of a d35S::NLS-YFP-GUS construct in different Arabidopsis seedlings (A-H) after 36 hr cocultivation when 0.005% Silwet L-77 and bacteria of OD600 = 0.5 were used in the cocultivation medium. Scale bar = 0.5 mm. [file 1746-4811-5-6-S2.tiff]

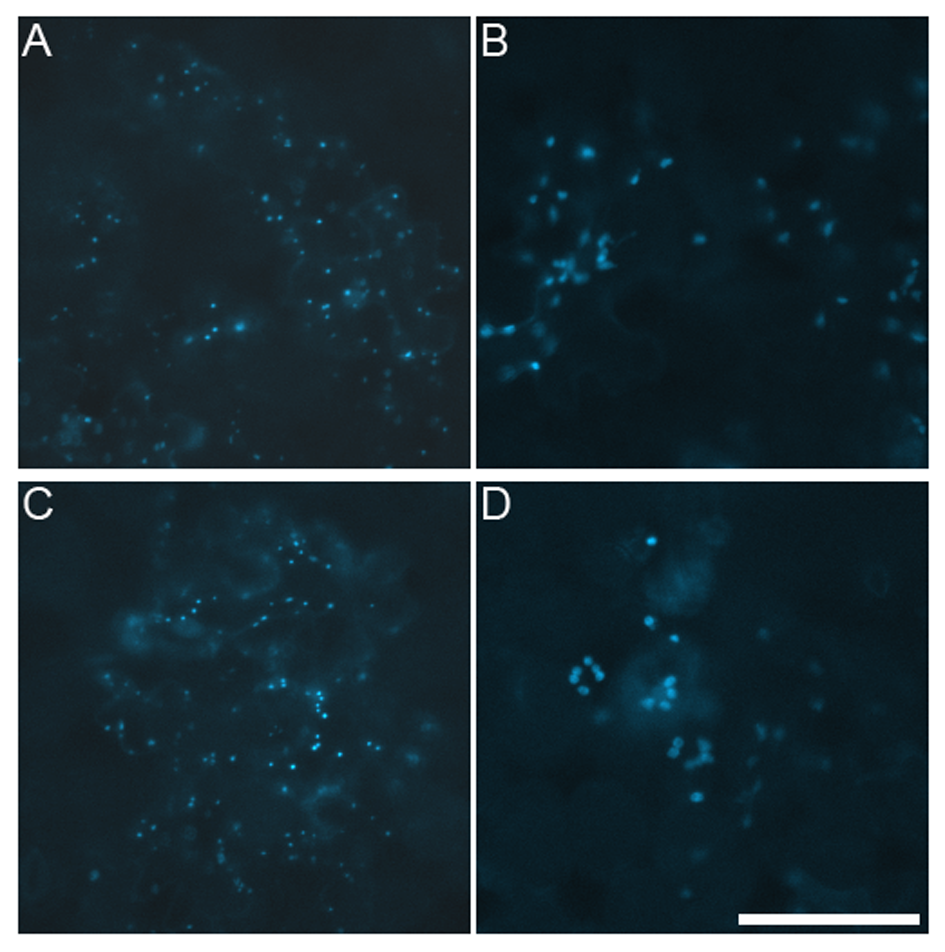

Supplement: Additional File 3 — Arabidopsis ecotype Col-0 seedlings and A. tumefaciens strain GV3101 in the FAST assay could be replaced by ecotype Ws seedlings and A. tumefaciens strain LBA4404, respectively. A, Arabidopsis ecotype Ws seedlings were cocultivated with A. tumefaciens strain GV3101 carrying d35S::Peroxisome-CFP marker. B, Arabidopsis ecotype Ws seedlings were cocultivated with A. tumefaciens strain GV3101 carrying d35S::Plastid-CFP marker. C, Arabidopsis ecotype Col-0 seedlings were cocultivated 37 with A. tumefaciens strain LBA4404 carrying d35S::Peroxisome-CFP marker. D, Arabidopsis ecotype Col-0 seedlings were cocultivated with A. tumefaciens strain LBA4404 carrying d35S::Plastid-CFP marker. Scale bar = 60 μm. [file 1746-4811-5-6-S3.tiff]

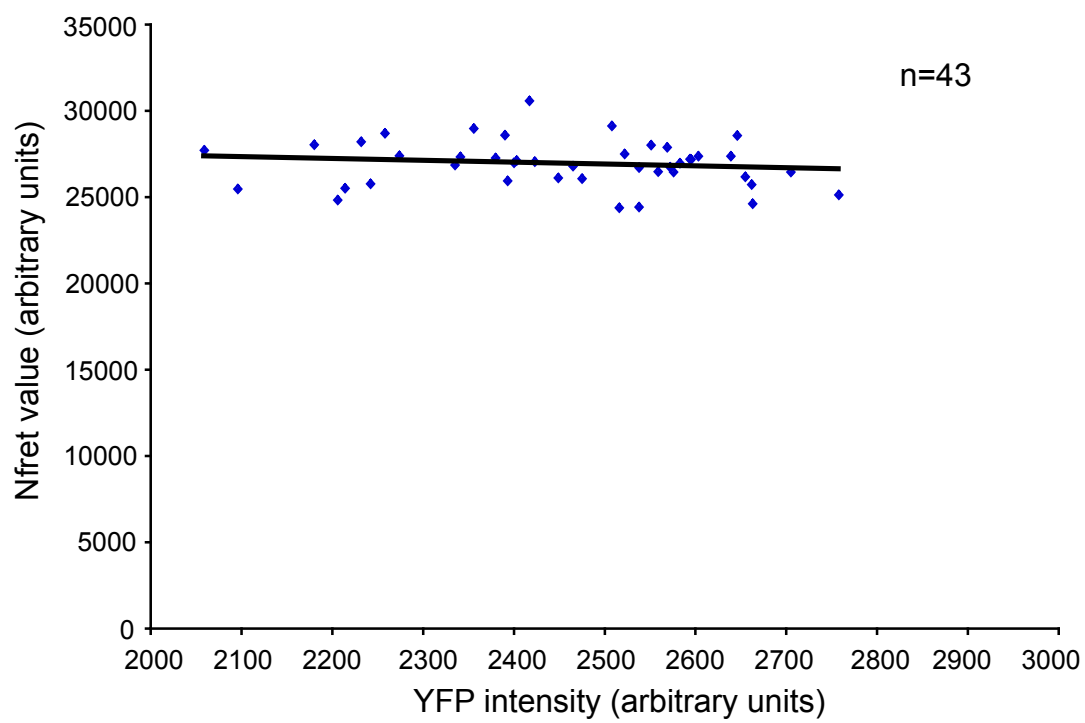

Supplement: Additional File 4 — Variation of transient expression levels in different cotyledon cells does not affect FRET quantification when Nfret algorithm was used. The FRET quantification by Nfret algorithm for 43 cells expressing YFP-Cerulean fusion showed that the Nfret value of these cells was almost constant irrespective of the variable cellular YFP concentration. Note that the regression line runs parallel to the x-axis. [file 1746-4811-5-6-S4.pdf]
